# Dual function of polycomb group proteins in differentiated murine T helper (CD4^+^) cells

**DOI:** 10.1186/1750-2187-6-5

**Published:** 2011-05-30

**Authors:** Eyal Jacob, Reut Hod-Dvorai, Or Lea Ben-Mordechai, Yulia Boyko, Orly Avni

**Affiliations:** 1 Department of Immunology, Rappaport Faculty of Medicine, Technion-Israel Institute of Technology, Haifa 31096, Israel

**Keywords:** *Il4*, *Ifng*, T helper cells, polycomb, transcription factors: NFAT, T-bet

## Abstract

**Background:**

Following antigen recognition, naive T helper (Th; CD4^+^) cells can differentiate toward one of several effector lineages such as Th1 and Th2; each expressing distinctive transcriptional profiles of cytokine genes. These cytokines eventually instruct the strategy of the immune response. In our search for factors that propagate the transcriptional programs of differentiated Th cells, we previously found that Polycomb group (PcG) proteins, which are known as epigenetic regulators that maintain repressive chromatin states, bind differentially the signature cytokine genes. Unexpectedly, their binding to the *Ifng *(*Interferon-g) *in Th1 cells and *Il4 *(*Interleukin-4*) in Th2 cells, was correlated with transcriptional activation. Therefore, in this study we aimed to determine the functional role of PcG proteins in the regulation of the expression of the signature cytokine genes.

**Methods:**

PcG proteins were knocked down in primary and established murine Th cells using transduction of lentiviruses encoding short hairpin RNAs (shRNAs) directed to Mel-18, Ezh2, Eed and Ring1A, representative of two different PcG complexes. The chromatin structure and the binding activity of PcG proteins and transcription factors at the *Ifng *promoter were assessed by chromatin immunoprecipitation (ChIP) assays.

**Results:**

Downregulation of PcG proteins was consistent with their function as positive regulators of the signature cytokine genes in primary and established Th1 and Th2 cells. Moreover, the PcG protein Mel-18 was necessary to recruit the Th1-lineage specifying transcription factor T-bet, and the T cell receptor (TCR)-inducible transcription factor NFAT1 to the *Ifng *promoter in Th1 cells. Nevertheless, our results suggest that PcG proteins can function also as conventional transcriptional repressors in Th cells of their known target the *Hoxa7 *gene.

**Conclusions:**

Our data support a model whereby the non-differentially expressed PcG proteins are recruited in a Th-lineage specific manner to their target genes to enforce the maintenance of specific transcriptional programs as transcriptional repressors or activators. Although our results suggest a direct effect of PcG proteins in the regulation of cytokine gene expression, indirect functions cannot be excluded.

## Background

When naive Th cells encounter an antigen for the first time, they can differentiate into the effector lineages Th1, Th2 and Th17 that differentially express cytokine genes [1-3]. The Th1 and Th2 lineages are characterized by the expression of the signature cytokines IFNγ and IL-4, respectively. IFNγ exerts protective functions in microbial infections and is observed clinically in cases of autoimmune diseases. IL-4 is strongly apparent in parasitic infections, and is associated with allergic reactions. The polarization of Th cells is most efficiently promoted by the cytokine milieu; IL-12 strongly potentiates the differentiation toward the Th1 lineage and IL-4 toward Th2. Although the activities of the polarizing cytokines ultimately lead to distinct Th phenotypes, the differentiation processes have similar features such as the expression of lineage-specifying transcription factors. The lineage-specifying transcription factors, T-bet in Th1 and GATA3 in Th2 cells, function as master regulators that establish the appropriate gene expression profiles for one lineage and oppose the alternative fates. Differential pattern of cytokine gene expression is also associated with selective recruitment of TCR-inducible transcription factors such as NFAT [4]. All of these activities are accompanied by major epigenetic changes [1-3] that are probably involved in the heritability of the transcriptional programs of differentiated Th cells. However, epigenetic regulation of Th cells preserves a certain level of plasticity that may allow adaptation to new immunological challenges [3,5-12].

Which factors are necessary to maintain the cellular memory of differentiated Th lineages? Several studies showed that the maintenance of the *Ifng *and *Il4 *transcriptional patterns in Th cells is not mediated exclusively by the lineage-specifying transcription factors [13-17]. Therefore, the lineage-specifying transcription factors may induce the expression of downstream specific factors or alternatively, facilitate restricted binding of a generally expressed epigenetic machinery. We previously showed that PcG proteins, whose role in maintaining gene silencing during embryogenesis is well known [18-24], bind the cytokine genes [25]. However, their binding activity was associated unconventionally with gene expression not silencing; in each lineage they were associated with the signature cytokine gene *Ifng *in Th1 cells and *Il4 *in Th2 cells [25].

The PcG and trithorax group proteins were first identified in *Drosophila*, as transcriptional regulators of the homeotic (Hox) genes during development. In contrast to the PcG proteins, the trithorax group proteins were characterized by their ability to maintain active transcription. The PcG proteins form two major complexes, PcG repressive complex 1 (PRC1), which contains the core proteins M33, Bmi-1, Mel-18, Ring1A, and Ring1B, and PRC2, with the core proteins Suz12, Ezh2, and Eed. However, biochemical purification demonstrated a significant variety in the content of these complexes [21,24,26]. The mechanisms by which PcG proteins mediate the epigenetic inheritance of transcriptional silencing are not fully understood [27], but they are known to be associated with histone modifications: Ring1B is histone H2A ubiquitin E3 ligase, and Ezh2 is histone methyltransferase that preferentially trimethylates histone H3 on lysine 27 (H3K27me3). The function of the PcG proteins also entails non-catalytic activities [26,28-30], such as chromatin compensation, long-range intrachromosomal interactions [31-35] and repression of transcriptional elongation [36]. Genome-wide binding profiles in *Drosophila*, murine and human embryonic stem cells have demonstrated that the PcG proteins have additional targets to the *Hox *genes, most of them are transcriptional regulators of development [37-42]. These genes are predominantly inactive in embryonic stem cells, and many are marked bivalently by both the PcG repressive mark H3K27me3 and the trithorax group permissive mark H3K4me3 [21,28,43]. Under this status, premature differentiation is prevented, and the pluripotency of the embryonic stem cells is maintained. Developmental signals eventually induce or stably silence the expression of these genes. The PcG proteins are also crucial during hematopoiesis [24,44,45], and their dysregulation has been linked with various human cancers, apparently due to the altered transcription of PcG-target genes that control cell proliferation and differentiation [24,46].

Considering the known function of the PcG proteins as transcriptional repressors, our previous results demonstrating the binding pattern of PcG proteins at the cytokine promoters in Th cells [25], raised the feasible scenario in which PcG proteins restrain overexpression of the active/accessible cytokine genes. However, our knockdown experiments are more compatible with the idea that the PcG proteins Mel-18, Ezh2, Eed and Ring1A can function as transcriptional activators of *Ifng *in Th1 and *Il4 *in Th2 cells, rather than transcriptional repressors. Moreover, in Th1 cells, Mel-18 was required for the recruitment of the crucial transcription factors T-bet and NFAT1 to the *Ifng *promoter. Nevertheless, our results also demonstrated that PcG proteins function as repressors of *Hoxa7 *in both Th1 and Th2 lineages, and of *Tbx21 *(which encodes T-bet) in Th2 cells. All together, our data propose a bi-functional role for the PcG proteins in differentiated Th cells.

## Methods

### Ethic statement

The studies have been reviewed and approved by the Inspection Committee on the Constitution of the Animal Experimentation at the Technion.

### Mice

Female BALB/c mice were purchased from Harlan Biotech, Israel, and maintained under pathogen-free conditions in the animal facility of the Faculty of Medicine, Technion-Israel Institute of Technology.

### In vitro Th-cell differentiation and Th-cell lines

CD4^+ ^T cells were purified from the spleen and lymph nodes of 3-4-week-old mice with magnetic beads (Dynal). For Th differentiation, the cells were stimulated with 1 μg/ml anti-CD3ε (to activate the TCR; 145.2C11, hybridoma supernatant) and 1 μg/ml anti-CD28 (to activate co-stimulatory molecule; 37.51, BioLegend) in Dulbecco's modified Eagle's medium (DMEM) supplemented with 10% fetal calf serum, L-glutamine, penicillin-streptomycin, nonessential amino acids, sodium pyruvate, vitamins, HEPES and 2-mercaptoethanol, in a flask coated with 0.3 mg/ml goat anti-hamster antibodies (ICN). For Th1 differentiation, the cells were stimulated in the presence of 10 ng/ml recombinant mouse IL-12 (R&D Systems) and 10 μg/ml purified anti-IL-4 antibodies (11B11). For Th2 differentiation, the cells were stimulated in the presence of 1000 U/ml mouse IL-4 (added as a supernatant of the 13L6 cell line), 5 μg/ml purified anti-IFNγ antibodies (XMG1.2), and 3 μg/ml purified anti-IL-12 antibodies (C178). After 2 days, the medium was expanded (fourfold) in the absence of anti-TCR or anti-CD28 antibodies, but in the continued presence of cytokines and other antibodies, which included 12 U/ml IL-2. The medium was then expanded every other day. After 6 or 8 days, the differentiated Th cells were left unstimulated or were restimulated with either PMA (15 nM) and ionomycin (0.75 μM) (P+I) or with anti-CD3ε and anti-CD28 antibodies, as indicated. When indicated, 2 μM Cyclosporine A (CsA) was added 0.5 hour before stimulation. The murine Th1 cell clone D5 (Ar-5 [47]) and Th2 cell clone D10 (D10.G4.1 [48]) were restimulated every 4-6 weeks with antigen (arsonate-conjugated ovalbumin for D5, and conalbumin for D10) in the presence of compatible CAF1/J mouse splenocytes that had been irradiated with 2000 rads.

### Chromatin Immunoprecipitation (ChIP)

The ChIP analysis was carried out as previously described [25]. Quantitative PCR was performed using Absolute Blue SYBR-Green ROX mix (Thermo Scientific, ABgene), according to the manufacturer's instructions, and an ABI Prism 7000 Sequence Detection System (Applied Biosystems) or Corbett Rotor gene 6000 (Qiagen). The dissociation curves after amplification showed that all the primer pairs generated single products. The amount of PCR product amplified was calculated relative to a standard curve of the input. The following antibodies were used: anti-Mel-18 (Santa Cruz; sc-8905), anti-ENX-1 (Ezh2, Santa Cruz; sc-17270 and 17268), anti-YY1 (Santa Cruz; sc-1703), anti-T-bet (Santa Cruz; sc-21003), anti-trimethyl-Histone H3(Lys4) (Upstate 07-473), anti-acetyl-Histone H3 (Upstate 06-599), anti-H3K27 (Upstate 07-449), and rabbit anti-NFAT1 (T2B1 and 67.1 [49]). The following primer sets were used: *Ifng *Promoter: 5'-CTGTGCTGTGCTCTGTGGAT-3' and 5'-GTGCCATTCTTGTGGGATTC-3'. *Il4 *Promoter: 5'-CTCCTGGAAGAGAGGTGCTG-3' and 5'-GTTGCTGAAACCAAGGGAAA-3'. *Hoxa7 *Exon 1: 5'-GCGGACAGGTTACAGAG -3' and 5'-CCCCGACAACCTCATACC-3'. *Tbx2*1 Promoter:5'-TTTCTCTCCCCGAGGAAGT-3' and 5'-AGGCGTGAGAATGCTCAG-3'. *Gata3 *distal promoter (1a):5'-TGCCTATGATAATGGCCCATTC-3' and 5'CTGCTCCTGGTGCCTACAAAG-3`. Gata3 proximal promoter (1b):5'-AAACGTTCTGGCTTGAATCCT-3' and 5'AGATTATTCCGTACGAGTGA-3'.

### RNA interference

The knockdown was accomplished with lentiviral shRNA (MISSION, Sigma). The lentiviral particles were produced by the calcium chloride-mediated transfection of HEK-293T cells. The supernatants were collected 24 hours post-transfection for 8 hrs and used immediately for transductions. For naïve Th-cell transduction, CD4+ cells were isolated and incubated in 6-well plates coated with anti-hamster antibodies, viruses, polybrene (8 μg/ml), and anti-CD3 and CD28 antibodies under skewing conditions for 16-18 hours. The medium was then replaced with fresh skewing medium, and 24 hours later, the medium was replaced again with selection medium, containing puromycin (8 μg/ml, Sigma) for 3 more days. The D5 and D10 cells were stimulated with antigen and transduced 24 hours later with the lentiviral particles for 16-18 hours, then the medium was replaced with medium for selection (IL-2 and puromycin) for 2-5 weeks. The following shRNA sequences were used: Ezh2; (Ez1) CGGCTCCTCTAACCATGTTTA, (Ez2) CCGCAGAAGAACTGAAAGAAA, (Ez3) GCTAGGCTAATTGGGACCAA. Mel-18; (M1) CGCTACTTGGAGACCAACAAA, (M2) CAAAGTTCCTCCGCAACAAA, (M3) ACCCTCTCCTTCCGCAGCCAT. Eed; (Ee1) TCTTGCTAGTAAGGGCACATA, (Ee2) CGGCTATTCGACAAACCAGTT, (Ee3) CCGGCCAGTGTGACATTTGGT. Ring1A; (R1) GCCTGGAAGGTGTCAGCGA, (R2) GTACGTGAAGACTACTGGG, (R3) CACTGACCTTGGAGCTTGT. Control scrambled shRNA; CAACAAGATGAAGAGCACCAA.

### RNA extraction and Real-time PCR

Total RNA was extracted, reverse-transcribed, and amplified. Melt curves were run to ensure amplification of a single product. The ratio between the transcripts was calculated as:

(1) ΔΔCt = [(Ct_(gene of interest) _-Ct_(β2 m)_)_*PcG*_-(Ct_(gene of interest) _-Ct_(β2 m)_)_*normalaizer*_]

(2) Fold increase = 2^- ΔΔCt^

*PcG *refers to the results obtained with PcG shRNAs as indicated and *normalaizer *with scrambled shRNA. The following primer sets were used: *Mel-18: *5'-AGCTGAACCCTCACCTCATGTG-3' and 5'-TACGATGCAGGTTTTGCAGAAG-3'. *Ezh-2: *5'-AGTCGCCTCGGTGCCTATAAT-3' and 5'-AAAGTGCCATCCTGATCCAGA-3'. *Eed*: 5'-ATCATAACCAGCCATTGTTTGGA-3' and 5'-GCAATAACCGTATCTCCCCCTG-3'. *Ring1A: *5'-CGCTGAATGGATCACTGACCT-3' and 5'-CCCCTTGTGACATCATTTTGG-3'. Beta-2-microglobulin: 5'-TTCTGGTGCTTGTCTCACTGA-3' and 5'CAGTATGTTCGGCTTCCCATTC-3'. *Ifng: *5'-GCGTCATTGAATCACACCTG-3' and 5'TGAGCTCATTGAATGCTTGG-3'. *Tbx21: *5'-GGTGTCTGGGAAGCTGAGAG-3' and 5'-GAAGGACAGGAATGGGAACA-3'. *Tnfa: *5'-CCAGACCCTCACACTCAGATCA-3' and 5'-CACTTGGTGGTTTGCTACGAC-3'. *Il13: *5'-ACCCAGAGGATATTGCATGGC-3' and 5'-CGTGGCGAAACAGTTGCTTT-3'. *Il4*: 5'-CCAAGGTGCTTCGCATATTT-3' and 5'-ATCGAAAAGCCCGAAAGAGT -3'. *Il5: *5'-CACCAGCTATGCATTGGAGA-3' and 5'-TCCTCGCCACACTTCTCTTT-3'. *Il10: *5'-CTGGACAACATACTGCTAACCG-3' and 5'-GGGCATCACTTCTACCAGGTAA-3'. *Rad-50: *5'-TGATAAGTTGTCTTGGGGTTTCC-3' and 5'-CTGTGTCTGACGCACCTGT-3'. *Hoxa7: *5'-GAAGCCAGTTTCCGCATCTA-3' and 5'-CGTCAGGTAGCGGTTGAAAT-3'. *Noxa: *5'-CCCACTCCTGGGAAAGTACA-3' and 5'- AAATCCCTTCAGCCCTTGAT-3'. *NFAT1: *5'-ACGGGAGTGACCGTCAAAC-3' and 5'-CGGGAGGGAGGTCCTGAAA-3'. *Gata3(1a): *5'-GAGCGTCAGCAACAGTGAAG-3' and 5'-CCACACTGCACACTGATTCC-3'. *Gata3(1b): *5'-CAATCTGACCGGGCAGGT-3' and 5'-CAGAGACGGTTGCTCTTCCG-3'

### Western Blot Analysis

Total protein was extracted using a Norgen kit (Cat 23000) or cytosolic/nuclear extract preparation, and the samples were separated by SDS polyacrylamide gel electrophoresis, transferred to PVDF membranes, and probed with anti-Ezh2 (612667, BD), anti-NFAT1 (67.1), anti-GATA3 (Santa Cruz; sc-268), anti-T-bet (Santa Cruz; sc-21003), anti-C-jun (Santa Cruz; sc-1694), or anti-α-Tubulin (Sigma; T-9026) antibodies.

### Flow Cytometry

Intracellular staining was performed using the BD Cytofix/Cytoperm kit, according to the manufacturer's instructions. The cells were stained with anti-Mel-18 (sc-10744, Santa Cruz) antibodies.

### Cytokine ELISA

The ELISA kits purchased from BioLegend were used.

## Results

### Mel-18 regulates transcriptional patterns in primary Th1 and Th2 cells

**Figure 1:**
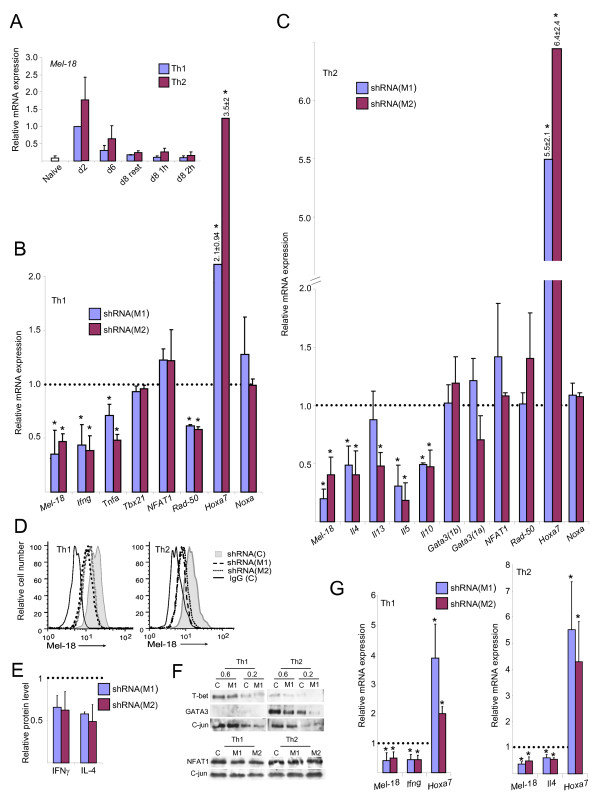
**Knockdown of Mel-18 downregulates the expression of cytokine genes in primary Th1 and Th2 cells**. (A) Quantitative RT-PCR for *Mel-18 *in naïve cells, differentiating Th1 and Th2 cells (days 2, 6, and 8), and 8^th^-day differentiated Th1 and Th2 cells after 1 or 2 hours of re-stimulation with P+I. The expression level in Th1 cells on day 2 was set as 1. (B,C) Quantitative RT-PCR for the indicated mRNAs following Mel-18 knockdown by *Mel-18*-directed shRNAs M1, M2 or control non-silencing scrambled shRNA during Th1 (B) and Th2 (C) differentiation. The results are presented relative to the control, defined as 1. The cells were transduced on day 0, and after 3 days of puromycin selection, on the 5^th ^day, they were re-stimulated with P+I for 2 hours. Differences between knockdown and control with p values ≤ 0.05 (Student's t test) are indicated with an asterisk. (D) Intracellular staining of Mel-18 in the indicated transduced Th1 and Th2 cells. (E) ELISA for the levels of IFNγ and IL-4 in the supernatants of 4-hour-stimulated (αCD3 and αCD28) Th1 and Th2 cells, respectively, after Mel-18 knockdown. The results with control shRNA were set as 1. (F) Western blot assessing the indicated proteins on the 5^th ^day of Th1 and Th2 cell transduced with control shRNA [C] or shRNA directed to Mel-18 [M1]. c-jun is used as loading control. (G) Quantitative RT-PCR for the indicated mRNAs after Mel-18 knockdown in Th1 (left) and Th2 (right) cells as described in Figure 1B, except the cells were re-stimulated with αCD3 and αCD28 antibodies. Differences between knockdown and control with p values ≤ 0.05 are indicated with an asterisk. The results in Figure 1 are the mean of two to five independent experiments +S.D., except in panels 1D and 1F, which are representative experiments.

Since, as we have previously shown, Mel-18 binds the *Ifng *promoter in Th1 cells and *Il4 *promoter in Th2 cells in association with gene expression [25], we wanted to examine its functional role in the regulation of these cytokine genes. Freshly isolated CD4^+ ^T cells (naive) were stimulated under Th1- or Th2-skewing conditions; Th1 cells expressed *Ifng *mRNA and Th2 cells expressed *Il4 *mRNA after stimulation, indicating that the cells were adequately differentiated and stimulated (data not shown). *Mel-18 *mRNA level increased in differentiating Th1 and Th2 cells, peaking on the second day (Figure 1A). The expression was basically unchanged following re-stimulation with PMA and ionomycin (P+I), which mimics TCR stimulation. Although the expression was higher in Th2 than in Th1 cells, the pattern was similar, supporting the idea that Mel-18 has parallel functions in both lineages.

Naive cells were stimulated under Th1- or Th2-skewing conditions, and simultaneously transduced with lentivirus encoding either one of two different shRNAs directed to *Mel-18 *or a control scrambled sequence (Figure 1B and 1C). In Th1 cells the level of *Mel-18 *mRNA was downregulated to ~35-45% (Figure 1B); Mel-18 protein level was also reduced (Figure 1D).

Downregulation of Mel-18 resulted in a decreased amount of *Ifng *mRNA (Figure 1B), as well as of IFNγ protein (Figure 1E), indicating that Mel-18 is a positive regulator of *Ifng*. Robust expression of *Ifng *requires the activity of transcription factors downstream to the TCR and cytokine receptors. However, the decline in *Ifng *expression probably did not result from changes in the expression levels of the mRNA or protein of the Th1-lineage specifying transcription factor T-bet, as these levels were essentially unaffected (Figure 1B and 1F). Similarly, the amounts of *NFAT1 (NFATc2) *mRNA and protein were almost unchanged (Figure 1B and 1F). The mRNA level of another cytokine *Tnfa *was also reduced, as well as of *Rad50*, which is expressed in both Th1 and Th2 cells from the *Il4 *locus. The expression of *Noxa*, encoding a pro-apoptotic protein that is repressed in Th cells by the PcG protein Bmi-1, [50], was almost unchanged. In contrast, the expression of *Hoxa7*, a known PcG target gene during development, which is also involved in T-cell leukemia [51], was significantly upregulated.

In Th2 cells, the expression of *Mel-18 *was knocked down to ~20-40%, and consequently the amount of *Il4 *mRNA was reduced by half (Figure 1C). The decrease in the levels of Mel-18 and IL-4 was confirmed (Figure 1D and 1E). These results indicate that in Th2 cells, as in Th1 cells, Mel-18 positively regulates the expression of the hallmark cytokine gene. Mel-18 was also necessary for the expression of *Il10 *and two other Th2 cytokine genes that are expressed from the *Il4 *locus, *Il5 *and *Il13 *(the later was reduced significantly only with one of the Mel-18 shRNAs). The levels of the two transcripts of the Th2-lineage specifying transcription factor *Gata3 *(containing the alternative 1a and 1b exons [52]) were similar to the control, except that one of the shRNAs led to a decreased *Gata3(1a) *level. We did observe a reduced level of GATA3 protein (Figure 1F), thus it is possible that Mel-18, like Bmi-1 [53], is involved in the stabilization of GATA3. The mRNA amounts of *NFAT1, Rad-50*, and *Noxa *were comparable to the control. However, the expression of *Hoxa7 *was increased following Mel-18 knockdown, even more than in Th1 cells.

Similar results demonstrating the positive regulation of cytokine genes and negative regulation of *Hoxa7 *by Mel-18 were obtained when the cells were re-stimulated with anti-CD3 and anti-CD28 antibodies (Figure 1G). To determine whether Mel-18 is essential only for the initiation of cytokine gene expression or also functions in differentiating Th1 and Th2 cells, the cells were transduced with the lentiviral shRNAs 48 hours after the first stimulation; the results were comparable (data not shown). Given our previous data showing the inducible and selective binding pattern of Mel-18 at the signature cytokine gene loci in Th1 and Th2 cells [25], the above results suggest that Mel-18 can function unconventionally as transcriptional activator of *Ifng *in Th1 cells and of *Il4 *in Th2 cells. Other cytokine genes, such as *Tnfa, Il5*, and *Il10*, may also be direct targets of Mel-18.

### Mel-18 has a dual function in Th cells

**Figure 2:**
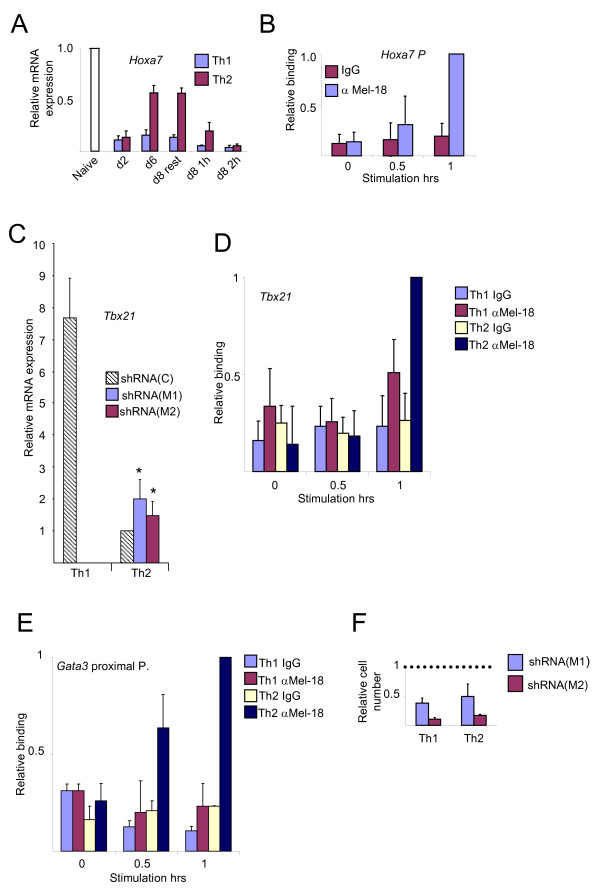
**Bi-functional role of Mel-18**. (A) Quantitative RT-PCR for *Hoxa7 *as described in Figure 1A. (B) ChIP experiment assessing the relative binding of Mel-18 at the *Hoxa7 *promoter region in resting and re-stimulated (P+I), 6-day-differentiated Th2 cells. The binding activity in 1-hr re-stimulated cells was set as 1. (C) Quantitative RT-PCR for *Tbx21 *mRNAs following the knockdown of Mel-18 as described in Figure 1B. The expression level of the control in Th2 cells was set as 1. (D) ChIP experiment assessing the binding of Mel-18 at the *Tbx21 *promoter in resting and re-stimulated (P+I), 6-day-differentiated Th1 and Th2 cells. The binding activity in 1-hr re-stimulated Th2 cells was set as 1. (E) ChIP experiment assessing the binding of Mel-18 at the *Gata3 *proximal promoter in resting and re-stimulated (P+I), 6-day-differentiated, Th1 and Th2 cells. The binding activity in 1-hr re-stimulated Th2 cells was set as 1. Similar results were obtained with the distal promoter. (F) The number of live cells on the 5^th ^day after Mel-18 knockdown relative to the control that was set as 1. The results in Figure 2 are the mean of two or three independent experiments +S.D.

We next aimed to test whether *Hoxa7 *is a direct target of Mel-18 in Th cells. The *Hoxa7 *mRNA level was high in normal naive cells and decreased after first stimulation and again after re-stimulation (Figure 2A). This pattern was the opposite to the pattern of cytokine gene expression. The expression of *Hoxa7 *was higher in Th2 cells than in Th1 cells, but the dynamic was similar. ChIP experiments confirmed that Mel-18 bound directly to the promoter region of *Hoxa7 *in Th2 cells (Figure 2B), and to a lesser extent, in Th1 cells (data not shown). The binding was induced following re-stimulation, as at the cytokine genes.

Since Mel-18 positively regulates the expression of cytokine genes and negatively of *Hoxa7*, it may function as both a transcriptional activator and repressor in Th cells, in a gene-specific manner. We previously observed some low binding activity of the PcG proteins at the opposing hallmark cytokine genes [25], and we asked whether Mel-18 represses their expression. The knockdown of *Mel-18 *concomitant with the first stimulation did not elevate the expression of *Ifng *in Th2 cells, which stayed low (data not shown). In contrast, the level of *Tbx21 *was increased in Th2 cells by ~1.5- 2-fold following downregulation of *Mel-18 *(Figure 2C). Mel-18 bound to the *Tbx21 *promoter in 1 hr re-stimulated Th1 cells, but its binding activity was much stronger in re-stimulated Th2 cells (Figure 2D). Thus, the expression of *Tbx21 *in Th2 cells was possibly repressed directly by Mel-18. All together these results suggest that Mel-18 plays a bi-functional role in Th cells.

The levels of *Il4, Il5*, and *Il13 *and of both *Gata3 *transcripts, were almost unaffected in Th1 cells (data not shown). In contrast to its binding pattern at *Tbx21*, Mel-18 was bound to the *Gata3 *promoters in correlation with gene expression, in Th2 and not in Th1 cells (Figure 2E). These results suggest that Mel-18 is not involved in the transcriptional silencing of *Gata3 *in Th1 cells, but it is possible that a more severe downregulation of Mel-18 is necessary to reveal its effect on *Gata3 *expression in Th2 cells, as in *Mel-18 *deficient mice [54].

Mel-18 knockdown resulted in ~20-50% of live cells compared to the control (Figure 2F), thus, in Th cells Mel-18 most likely regulates the expression of additional genes regulating proliferation or cell survival.

### Eed and Ring1A positively regulate the expression of the hallmark cytokine genes in Th cells

We next investigated the function of the PRC2 protein, Ezh2, which also binds to cytokine gene promoters selectively in Th cells [25]. The knockdown of *Ezh2 *in Th2 cells resulted in decreased expression of *Il4*, however, in Th1 cells the expression of *Ifng*, was unchanged (data not shown). At present, we do not know whether the knockdown was insufficient to reveal an effect, or whether Ezh2 is unnecessary for the transcription of *Ifng *in developing Th1 cells. Alternatively, Ezh2 function is partially redundant with Ezh1 [55,56].

**Figure 3:**
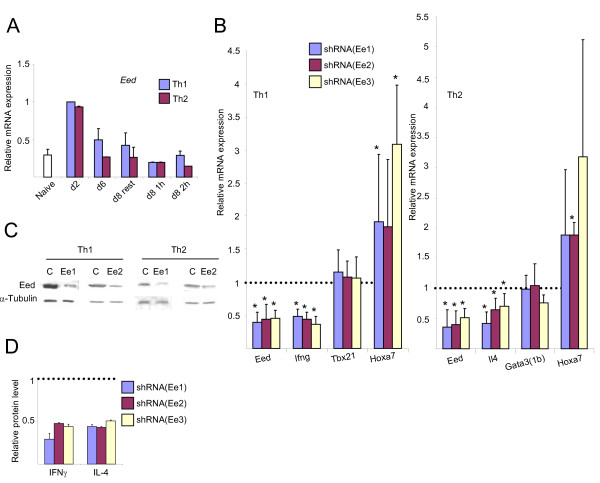
**Knockdown of Eed downregulates the expression of cytokine genes in primary Th1 and Th2 cells**. (A) Quantitative RT-PCR for *Eed*, as described in Figure 1A. (B) Quantitative RT-PCR for the indicated mRNAs after Eed knockdown in Th1 (left) and Th2 (right) cells with *Eed-*directed shRNAs Ee1, Ee2 and Ee3 or non-silencing scrambled shRNA as a control. The cells were transduced on day 0 and re-stimulated on the 5^th ^day (αCD3 and αCD28) for 2 hours. The results are presented relative to the control, defined as 1. Differences between knockdown and control with p values ≤ 0.05 are indicated with an asterisk. C. Western blot for Eed for the indicated transduced Th1 and Th2 cells following knockdown of Eed. D. ELISA for the levels of IFNγ and IL-4 in the supernatants of 2-hour-stimulated (αCD3 and αCD28) Th1 and Th2 cells, respectively, after Eed knockdown. The results with control shRNA were set as 1. The results in Figure 3 are the mean of three to five independent experiments +S.D., except in panel 3C, which is a representative experiment.

To determine whether other PRC2 proteins regulate cytokine gene expression in Th1 cells, we repeated the experiments using shRNAs directed to Eed. Eed, as other PcG proteins, binds differentially to *Il4 *and *Ifng *promoters, in correlation with gene expression [25]. Eed is necessary for the histone lysine methyltransferase activity of Ezh2, and also for the propagation of the H3K27me3 mark [57,58]. The pattern of *Eed *mRNA expression in Th cells (Figure 3A) was similar to that of *Mel-18 *mRNA (Figure 1A).

Three different shRNAs knocked *Eed *down to ~40-50% in Th1 cells and reduced the expression of *Ifng *to the same extent; in contrast, the expression of *Tbx21 *was unchanged, and that of *Hoxa7 *increased (Figure 3B, left panel). Similarly, in Th2 cells, three different shRNAs knocked *Eed *down and consequently the expression of *Il4 *was decreased, but not that of *Gata3(1b)*, at least with two of the shRNAs (Figure 3B, right panel). The expression of *Hoxa7 *was always higher following Eed knockdown, but the results in some cases are not considered statistically significant because of large differences in the extent of the upregulation (For example, downregulation of Eed with shRNA (Ee3), increased the expression of *Hoxa7 *mRNA by 1.51, 4.28, 5.34, and 1.55 in four different experiments). The decreased expression of Eed protein was confirmed (Figure 3C), as well as of IFNγ and IL-4 (Figure 3D). These results indicate that PRC2 components are necessary for the expression of the signature cytokine genes in developing Th1 and Th2 cells.

**Figure 4:**
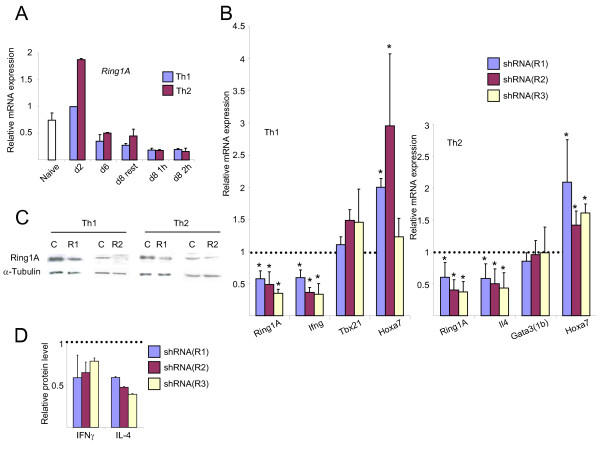
**Knockdown of Ring1A downregulates the expression of cytokine genes in primary Th1 and Th2 cells**. The experiments as described in Figure 3 were performed for Ring1A.

The PRC1 complex contains also Ring1A, which binds the cytokine genes in correlation with their expression [25]. Ring1B catalyzes the ubiquitination of histone H2A (H2AK119ub1), while both Ring1A and Ring1B contribute to this activity *in vivo *[59-61]. This modification can repress gene expression by maintaining RNA polymerase II at poised configuration [36]. The pattern of *Ring1A *expression resembled those of the other PcG proteins, although its relative level was higher in naive cells (Figure 4A). The knockdown of *Ring1A *in Th1 and Th2 cells with each one of three different shRNAs downregulated its expression approximately by half (Figure 4B). Accordingly, the expression of *Ifng *in Th1 and *Il4 *in Th2 cells was reduced in a similar degree, but that of the lineage-specifying transcription factors was not. The expression of *Hoxa7 *was increased in both Th1 and Th2 cells with most of the shRNAs. Decreased amounts of the protein Ring1A and of the proteins IFNγ and IL-4 was also observed (Figure 4 C,D). The knockdown of Eed or Ring1A, like that of Mel-18, reduced the number of live cells (data not shown). Under these conditions, none of the signature cytokines or tissue-specifying transcription factors was derepressed in the opposing lineage following the knockdown of Eed or Ring1A (data not shown). Taken together, our results show that PcG proteins from two different complexes positively regulate the expression of the hallmark cytokine genes in Th1 and Th2 cells.

### PcG proteins positively regulate the expression of cytokine genes in established Th cells

**Figure 5:**
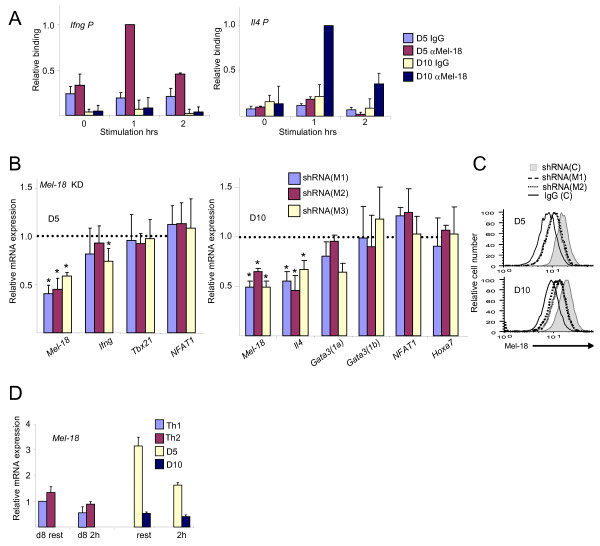
**Mel-18 is a positive regulator of cytokine genes in D5 and D10 cells**. (A) ChIP experiment assessing the binding of Mel-18 at the *Ifng *(left) and *Il4 *(right) promoters in D5 and D10 cells. The cells were stimulated with antigen-presenting cells (APCs) with the appropriate peptides, cultured for 2-3 weeks, and then left unstimulated or were re-stimulated (P+I) for the indicated time points. In the panel of the *Ifng *promoter the result of 1 hr stimulation of D5 cells was set as 1, and in the *Il4 *promoter panel the result of 1 hr stimulation in D10 was set as 1. (B) Quantitative RT-PCR for the indicated mRNAs following Mel-18 knockdown in D5 (left) and D10 (right) cells. The cells were re-stimulated with APCs with the appropriate peptides, transduced with lentiviral shRNAs, cultured in the presence of puromycin for 2-3 weeks, and then re-stimulated with anti-CD3 and anti-CD28 antibodies for 2 hours. The results are presented relative to the control, defined as 1. Differences between knockdown and control with p values ≤ 0.05 are indicated with an asterisk (C) Intracellular staining of Mel-18 in the indicated transduced D5 and D10 cells. (D) Quantitative RT-PCR for *Mel-18 *in resting and 2-hour-restimulated cells as indicated. The expression level in resting Th1 cells was set as 1. The results in Figure 5 are the mean of three to six independent experiments +S.D., except panel 5C, which is a representative experiment.

Since the PcG proteins are well known for their role in maintaining epigenetic states (although in association with gene silencing), we examined their effect on cytokine gene expression in established Th cells, using the murine D5 (Th1) and D10 (Th2) clones. Mel-18 bound to *Ifng *and *Il4 *genes in D5 and D10 cells in correlation with their expression, as in primary Th1 and Th2 cells (Figure 5A). In D5 cells, *Mel-18 *mRNA was knocked down to ~50% (Figure 5B, left panel), and Mel-18 protein was reduced as well (Figure 5C). The level of the *Ifng *mRNA varied; it was reduced to about half in some experiments and unchanged in others (for example, the results for shRNA (M1) were: 0.62, 0.53, 0.52, 1.07, 1.19, 0.97). The knockdown was probably not efficient enough to consistently reduce the mRNA levels, especially since D5 cells express high levels of *Mel-18 *mRNA (Figure 5D). The levels of the *Tbx21 *and *NFAT1 *mRNAs were basically unaffected. The D5 cells expressed very low amounts of *Hoxa7 *mRNA (data not shown). In D10 cells, the expression of *Mel-18 *mRNA and protein were reduced by ~50% (Figure 5B, right panel and Figure 5C), and consequently, the *Il4 *mRNA level was diminished. The level of *Gata3(1a) *was reduced moderately with two shRNAs, and those of *Gata3(1b) *and *NFAT *were almost unchanged. In contrast to primary Th2 cells, the level of *Hoxa7 *was similar to the control, suggesting that in committed cells the expression of this gene is repressed by other, probably more permanent mechanisms.

**Figure 6:**
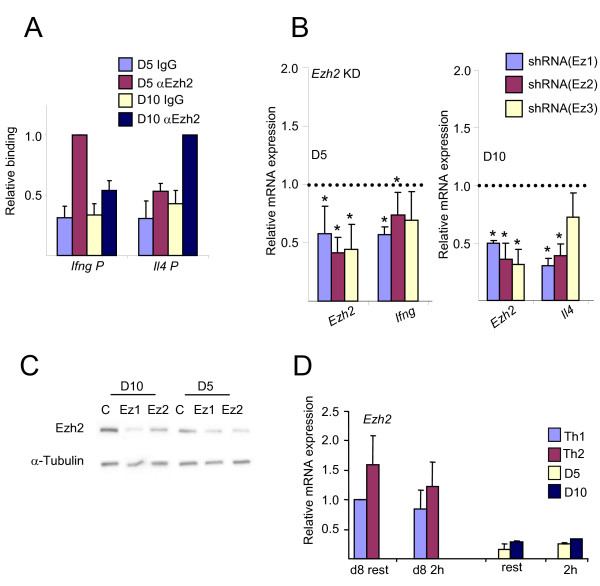
**Ezh2 is a positive regulator of cytokine genes in D5 and D10 cells**. (A) ChIP experiment assessing the binding of Ezh2 at the *Ifng *and *Il4 *promoters in stimulated D5 and D10 cells. In D5 cells the relative binding at the *Ifng *promoter was set as 1, and in D10 cells the relative binding to the *Il4 *promoter was set as 1. (B) Quantitative RT-PCR for the indicated mRNAs following Ezh2 knockdown in D5 (left) and D10 (right) cells. The cells were re-stimulated (αCD3 and αCD28) for 2 hours. The results are presented relative to the control, defined as 1. Differences between knockdown and control with p values ≤ 0.05 are indicated with an asterisk. (C) Western blot for Ezh2 for the indicated transduced D5 and D10 cells following knockdown of Ezh2. (D) Quantitative RT-PCR for *Ezh2 *in resting and 2-hour-restimulated cells as indicated. The expression level in resting Th1 cells was set as 1. The results in Figure 6 are the mean of three to six independent experiments +S.D., except panels 6C, which is a representative experiment.

The binding of Ezh2 at the cytokine promoters in stimulated D5 and D10 cells was also differential (Figure 6A), although not as strong as of Mel-18. Knockdown of *Ezh2 *mRNA (Figure 6B**) **and consequently of Ezh2 protein (Figure 6C), resulted in lower amounts of *Ifng *and *Il4 *mRNAs in D5 and D10 cells, respectively. The downregulation of *Ifng *expression following Ezh2 knockdown in D5 cells was in contrast to the results obtained with primary Th1 cells, and perhaps reflects the low *Ezh2 *mRNA level in these cells (Figure 6D), which may have caused the knockdown to have a greater impact. Alternatively, the difference may be related to the differentiation stage or to the possibility that Ezh2 is necessary to propagate the epigenetic state during cell cycle, and therefore a longer-term experiment was required to observe an effect. All together, these results demonstrate that PcG proteins are necessary for the expression of the signature cytokine genes, even in committed Th cells.

### Mel-18 is required for the binding of NFAT1 and T-bet to the Ifng promoter

**Figure 7:**
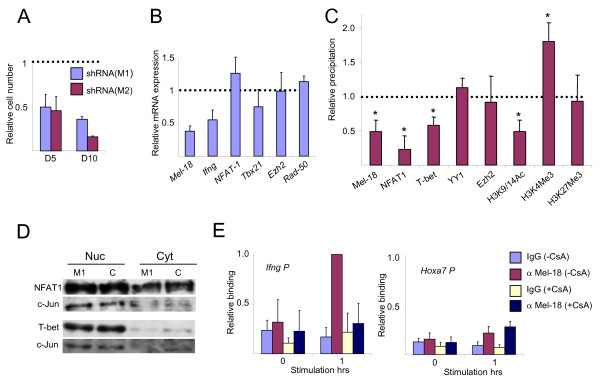
**Mel-18 is required for the recruitment of T-bet and NFAT to the *Ifng *promoter in D5 cells**. (A) The numbers of live cells following Mel-18 knockdown in D5 and D10 cells relative to the control that was set as 1. (B) Quantitative RT-PCR for the indicated mRNAs in 2-hour-restimulated (P+I) D5 cells following Mel-18 knockdown (3-4 weeks of selection) with shRNA M1 and control shRNA. The results are presented relative to the control that was set as 1. (C) ChIP experiment assessing selected modifications and the binding activity of the indicated factors at the *Ifng *promoter following Mel-18 knockdown as described in Figure 7B. Differences between knockdown and control with p values ≤ 0.05 are indicated with an asterisk. (D) Western blot assessing T-bet and NFAT1 under the conditions described in Figure 7B. c-jun is used as loading control. Cyt; cytosolic, Nuc; nuclear. (E) ChIP experiment assessing the binding of Mel-18 at the *Ifng *and *Hoxa7 *promoters in resting and 1-hour-restimulated D5 cells in the presence or absence of CsA. The binding activity at the *Ifng *promoter following stimulation in the absence of CsA was set as 1. The results in Figure 7 are the mean of three to five independent experiments +S.D., except panel 7D, which is a representative experiment.

To examine the mechanisms underlying PcG function, we next characterized the state of the *Ifng *promoter in *Mel-18*-knockdown cells. These experiments were done using D5 cells, since their proliferation was less sensitive to the downregulation of *Mel-18 *after 3-4 weeks of selection, compared with D10 cells (Figure 7A). The knockdown of Mel-18 in D5 cells with shRNA(M1) decreased the *Ifng *expression following re-stimulation with P+I, similar to but more consistent than that observed following re-stimulation with anti-CD3 and anti-CD28 antibodies (Figures 7B and 5B). A ChIP experiment demonstrated that, as expected, the downregulation of Mel-18 diminished significantly its binding to the *Ifng *promoter, but as a result also the binding of the transcription factors NFAT1 and T-bet (Figure 7C). This was probably a specific effect and not a general change in the locus accessibility, because the binding of the PcG protein YY1 was unaffected. The binding of Ezh2 was not significantly reduced either. This also did not reflect changes in the level of NFAT1 or T-bet (Figure 7D), as although the level of *T-bet *mRNA decreased moderately (Figure 7B), almost no change was detectable at the protein level.

The binding of Mel-18 at the *Ifng *promoter was abolished in D5 cells in the presence of Cyclosporin A (CsA), which impairs the translocation of NFAT to the nucleus (Figure 7E). We previously observed similar results in primary Th cells [25]. In contrast, the binding activity of Mel-18 at the *Hoxa7 *promoter region was unaffected. These results suggest that the binding activities of the PcG proteins and NFAT are mutually dependent, and also that the mechanisms for the recruitment of the PcG proteins differ, depending on their function.

The knockdown of Mel-18 resulted in a reduced acetylation of histone 3 (H3K9/14Ac), which is a transcriptional permissive mark (Figure 7C). It is not clear yet whether this was the cause or the consequence of the decreased binding activity of T-bet and NFAT. In contrast, H3K4me3, which is also a permissive mark, was stronger. We did not recognize significant changes in H3K27me3, which was lower at the *Ifng *promoter than at the *Il4 *promoter (Figure 7C and data not shown). We did not observe a detectable level of H2AK119ub1 before or after Mel-18 knockdown (data not shown). Taking together these results suggest that the activity of the PcG proteins as transcriptional activators does not necessarily involve their classical histone modifications.

## Discussion

While the role of the PcG proteins during development has been extensively studied, there is little information about their functions in differentiated cells. We used the RNAi approach to study the functional role of the PcG proteins in Th cells since the knockout of PRC2 members causes embryonic lethality [62]. And although the PRC1 members, excluding Ring1b, display more redundancy, they have a pivotal role during hematopoiesis [24,44,45].

*Mel-18 *deficient mice have severe proliferative defects in lymphoid cells resulting in hypoplasia of spleen and thymus [44,63,64], and have less than 5% of the thymocytes of wild-type mice [65]. The differentiation of *Mel-18*-deficient Th2 cells is impaired, but not of Th1 [54]. It is possible that the absence of Mel-18 during T cell development interferes selectively with Th2 differentiation. The expression of *Gata3 *is reduced in cells derived from *Mel-18*-deficient mice [54], and - apart from the fact that GATA3 is important for T cell development in the thymus [66] - its early presence may be required for normal subsequent Th differentiation. GATA3, for example, might be necessary for the initiation of the intrachromosomal conformation at the *Il4 *locus [67]. The negative regulation of *Tbx21 *by Mel-18 in Th2 cells, as we showed, may also explain the modest enhancement in the expression of IFNγ in Th cells derived from *Mel-18*-deficient mice.

T cells in general response moderately to the RNAi procedure [68], and since the knockdown is partial, our results probably underestimate the importance of PcG protein functions in Th cells. Nevertheless, our data suggest that PcG proteins from two different complexes can function as transcriptional activators of the signature cytokine genes in primary Th1 and Th2 cells. We showed similar results also in primary Th17 cells [69]. And Mel-18 and Ezh2 function as positive regulators of cytokine genes in established Th1 and Th2 cell lines. Therefore, PcG proteins may act as a general Th-machinery that induces the expression or maintains the permissive epigenetic state of the effector cytokine genes. The PcG proteins also perform the opposite activity in Th cells, acting as transcriptional repressors of *Hoxa7 *in both lineages, and of *Tbx21 *in Th2 cells. We also cannot exclude the possibility that a stronger downregulation of PcG proteins is necessary to detect negative regulation of the opposing cytokine genes. The PcG probably have many other targets in Th cells and the type of PcG activity might depend on the epigenetic context, the proteins available at the target genes, and differential posttranslational modifications. But it is also possible that discrete PcG complexes or isoforms are involved.

Since we observed fewer live cells following the knockdown of Mel-18, Eed, or Ring1A, additional targets of these PcG proteins in Th cells are probably genes involved in proliferation or survival. It was shown that the PcG proteins Bmi-1 and Ring1B modulate apoptosis of Th2 cells through regulation of *Bim *and *Noxa *genes, respectively [50,70]. However, the lower cell numbers is probably not the reason for the downregulation in the expression of the cytokine genes based on several reasons: (i) The expression of the mRNAs of NFAT and the lineage specifying transcription factors was in general normal, indicating that the effect on the cytokine genes was specific and did not reflect a general impairment in lineage differentiation or gene expression; (ii) There was no correlation between the potential of specific shRNAs to reduce the expression of cytokines and their effect on cell numbers; shRNA(M2) directed to Mel-18 reduced the cell numbers more efficiently than shRNA(M1), but both presented similar efficiencies in downregulating cytokine gene expression; (iii) knock down of Ezh2 in Th17 cells [69], and with some shRNAs in Th1 and Th2 cells (data not shown) did not result in diminished cell numbers, but yet downregulated cytokine gene expression.

Because the PcG proteins are well known as transcriptional silencers, one possible explanation for our results is that the PcG proteins positively regulate the expression of the cytokine genes indirectly, by repressing a repressor(s). However, it is more likely that the PcG proteins function as genuine transcriptional activators of cytokine genes for the following reasons: (i) The PcG proteins bind directly, differentially, and inducibly to the active cytokine genes [25]. It is less plausible that such a strong correlation is unrelated to their function. Moreover, their binding pattern was dynamic following stimulation, and different PcG proteins bind regulatory elements of the same gene with differential relative intensity [25]. Also, the PcG proteins do not bind exclusively active genes; for example, Mel-18 was associated with *Tbx21 *in Th2 stronger than in Th1 cells, in association with gene repression. All together these results strengthen the idea that the binding activity is specific and does not reflect irrelevant 'stickiness' to accessible DNA; (ii) Several studies in *Drosophila *and in mammalian embryonic stem cells reported that 10-20% of the PcG targets were transcriptionally active; therefore, the binding of PcG proteins does not necessarily lead to silencing (reviewed in [43]). Moreover, in a human colon cell line, Suz12 and Ezh2 were associated with the promoters of several genes that were downregulated upon Suz12 depletion [71]. In addition, Ezh2 (but not other PcG proteins) was found as a transcriptional activator of *c-Myc *and *cyclin D1 *in a breast cancer cell line [72]. The dual function of the PcG proteins may be a dominant feature of more-committed cells, but possibly exists in other developmental stages as well.

Recently, Th cells were shown to exhibit more plasticity than it was previously appreciated [3,5-12]. Th17 cells exhibit the highest degree of flexibility, even at a late developmental stage, in the absence of polarizing cytokines [73]. However, viral infection can reprogram also Th2 cells *in vivo *into a Th2-Th1 cells expressing GATA3 and T-bet simultaneously [74]. Whereas *Tbx21 *in Th1 cells and *Gata3 *in Th2 cells are marked selectively with the permissive H3K4me3 and not with the repressive H3K27me3, in the opposing lineage and in Th17 cells, these genes are marked bivalently [75], like the transcriptional regulator genes during embryonic development. This is considered as a "poised" gene status, with the potential for subsequent activation or silencing. *Tbx21 *was bound more strongly by Mel-18 in Th2 than Th1 cells, in correlation with gene repression. Indeed its expression was derepressed in Th2 cells following knockdown of Mel-18. These results suggest that the PcG proteins may regulate, as transcriptional activators or repressors, gene networks that specify the expression profiles in Th cells. *Gata3 *expression was not increased following the knockdown of Mel-18 in Th1 cells, and it is possible therefore that it is silenced by other, more stable, mechanisms, or that the knockdown strength was not sufficient to observe this effect.

We found that PcG proteins were necessary during early Th differentiation and in established Th cell lines. They were bound under resting conditions, but their binding activity was increased following TCR stimulation, in correlation with the inducible expression of the cytokines (here and [25]). Therefore, they might function as either: (i) acute transcription factors similar to NFAT or even as co-factors; (ii) epigenetic regulators that inducibly increase their binding activity to propagate the transcriptional status during cell duplication, which normally follows stimulation. They may cooperate with other chromatin remodeling factors such as BRG1, which is also recruited in a selective and partially inducible manner to the cytokine genes *Ifng *in Th1 [76] and *Il4 *in Th2 cells [77,78]. Also they may interact with trithorax group proteins such as MLL that was found to be essential for the ability of Th2 memory cells to express the Th2 cytokines [79].

In Th2 cells, the expression level of the GATA3 protein was decreased following Mel-18 knockdown. However, stabilization of the lineage specifying transcription factors cannot be the only mechanism by which the PcG proteins regulate cytokine gene expression. For example, the level of GATA3 was essentially unchanged following the knockdown of Ezh2 in Th2 cells (data not shown), but the expression of *Il4 *mRNA was reduced. Similarly, the expression of T-bet was unchanged in Th1 and D5 cells following the knockdown of Mel-18, but the expression of *Ifng *mRNA was downregulated.

What are the relative functional roles of the lineage specifying transcription factors, TCR-inducible transcription factors and the epigenetic machinery in the potential network that maintains the transcriptional programs in Th cells? The restricted recruitment of the generally expressed PcG proteins is probably regulated directly or indirectly by the selectively expressed lineage-specifying transcription factors. The targeting of the PcG proteins to the cytokine genes is also most likely NFAT-dependent since we found that impaired translocation of NFAT to the nucleus abrogates the binding of PcG proteins at the cytokine genes in primary [25] and established Th cell lines (here and data not shown). It is possible that NFAT is directly involved in the recruitment of these PcG proteins, and the same mechanisms that restrict the binding of NFAT [4], dictate the selective targeting of PcG proteins.

Conversely, we also showed that in an established Th1 cell line Mel-18 was necessary for the recruitment of T-bet and NFAT1 to the *Ifng *promoter. Therefore, the binding activities of the PcG proteins and of NFAT are mutually dependent. The binding activities of the PcG proteins and the lineage-specifying transcription factors can also be interrelated. The PcG proteins in general do not have sequence-specific binding sites, but they may stabilize the binding of these transcription factors on the chromatin. They may also have an indirect effect by affecting chromatin accessibility, although it must be specific since the association of YY1 was unchanged. Instead, PcG proteins can regulate the expression of factors that are necessary for the binding activity of T-bet and NFAT.

The lineage specifying transcription factors are not necessarily associated with the PcG proteins, but both types of factors can take functional turns in a way that the binding of each of them is a prerequisite for the binding of the other one. The lineage-specifying transcription factors may specify the binding sites for the general machinery, which on its turn facilitates the heritability of the epigenetic programs during mitosis, and by that assists the re-establishment of the specific factors. Absence of the specific factors that re-enforce the selective recruitment of the general maintenance machinery, may result in progressive dilution of epigenetic marks during mitosis, and consequently impaired the accessibility of the lineage specifying transcription factors to their target genes.

## Conclusions

Our results show that PcG proteins have a dual function in Th cells as positive and negative regulators of gene expression. Our results also suggest that both activities can results from a direct effect. The lineage-dependent recruitment of the PcG proteins and consequently their restricted regulation of the effector cytokine genes have a potential for therapeutic applications. The expression of Ezh2 mRNA is significantly downregulated in patients with active systemic lupus erythematosus [80], which could be one of the reasons for their abnormal cytokine production.

## List of abbreviations used

(PcG): Polycomb group; (Th): T helper; (shRNAs): short hairpin RNAs; (TCR): T cell receptor; (ChIP): chromatin immunoprecipitation

## Competing interests

The authors declare that they have no competing interests.

## Authors' contributions

OA designed the concept of the research and experiments and wrote the paper, EJ and RH-D designed and performed most of the experiments, OLB performed the knockdown experiments of Eed and Ring1A, and YB performed some of the knockdown experiments in the established Th cells. All authors read and approved the final manuscript.
